# A class of genes in the HER2 regulon that is poised for transcription in breast cancer cell lines and expressed in human breast tumors

**DOI:** 10.18632/oncotarget.2676

**Published:** 2014-11-04

**Authors:** Farah B. Rahmatpanah, Zhenyu Jia, Xin Chen, Jessica E. Char, Bozhao Men, Anna-Clara Franke, Frank E. Jones, Michael McClelland, Dan Mercola

**Affiliations:** ^1^ Department of Pathology and Laboratory Medicine, University of California, Irvine, CA, USA; ^2^ Department of Cell and Molecular Biology, Tulane University, New Orleans, Louisiana, USA; ^3^ Department of Microbiology and Molecular Genetics, University of California, Irvine, CA, USA; ^4^ Department of Statistics, University of Akron, Akron, Ohio, USA; ^5^ Department of Family and Community Medicine, Northeast Ohio Medical University, Rootstown, Ohio, USA

**Keywords:** RNA polymerase II, promoter array, HER2, breast cancer, expression analysis, HER2 Regulon, chromatin immunoprecipitation, mammospheres, mammary stem cells, NANOG, SOX2, OCT3/4

## Abstract

HER2-positive breast cancer accounts for 25% of all cases and has a poor prognosis. Although progress has been made in understanding signal transduction, little is known of how HER2 achieves gene regulation. We performed whole genome expression analysis on a HER2^+^ and HER2^−^ breast cancer cell lines and compared these results to expression in 812 primary tumors stratified by their HER2 expression level. Chip-on-chip with anti-RNA polymerase II was compared among breast cancer cell lines to identify genes that are potentially activated by HER2. The expression levels of these HER2-dependent POL II binding genes were determined for the 812 HER2+/− breast cancer tissues. Genes differentially expressed between HER2+/− cell lines were generally regulated in the same direction as in breast cancer tissues. We identified genes that had POLII binding in HER2^+^ cell lines, but without significant gene expression. Of 737 such genes “poised” for expression in cell lines, 113 genes were significantly differentially expressed in breast tumors in a HER2-dependent manner. Pathway analysis of these 113 genes revealed that a large group of genes were associated with stem cell and progenitor cell control as indicated by networks centered on NANOG, SOX2, OCT3/4. HER2 directs POL II binding to a large number of genes in breast cancer cells. A “poised” class of genes in HER2^+^ cell lines with POLII binding and low RNA expression but is differentially expressed in primary tumors, strongly suggests a role of the microenvironment and further suggests a role for stem cells proliferation in HER2-regulated breast cancer tissue.

## INTRODUCTION

HER2/ErbB2 is a trans-membrane tyrosine kinase of the EGF (Epidermal Growth Factor) receptor family. Amplification and expression of the HER2 oncogene in breast cancer occurs in 25-40%of cases, and is associated with aggressive disease with poor prognosis [1]. Extensive research has illuminated numerous potential mechanisms that account for its devastating effects in breast cancer. The receptor readily dimerizes leading to auto phosphorylation and activation of heterodimer partners. Heterodimer formation with the other three family members, EGFR, HER3 and HER4 is known [2-4]. In particular HER2/HER3 heterodimers stimulate multiple signal transduction pathways [5].

The activated receptors in turn recruit adaptor proteins which sequester substrates for downstream activation. HER2 signals through at least four major pathways including Map kinase, PI3K/Akt, Phospholipase C, and STAT. The MAP kinase path leads to the activation of genes that promote cell proliferation. PI3K/Akt promotes down regulation of several intermediates of apoptosis thereby promoting increased cell survival. Together these complimentary effects provide an attractive mechanism for the oncogenic role of HER2 [6].

An important discovery for the treatment of HER2 positive breast cancer was the demonstration that the humanized monoclonal antibody, Herceptin/Trastuzumab, directed against the extracellular portion of HER2 promoted a significant increase in survival in large scale human phase III clinical trials [7]. Indeed, the introduction of Herceptin ushered in the era of personalized or targeted cancer therapy. In addition, Herceptin synergizes with Pertuzumab, a humanized monoclonal antibody against an epitope of the extracellular domain of HER2 that is distinct from the target sites for Herceptin [8] and increases progression-free survival in metastatic HER2^+^ breast cancer by 6 months, which is beyond that observed by either agent alone. Pertuzumab, Lapatinib and combination therapies have improved the outlook for women with HER2-positive breast cancer [9]. However, none of these regimens are curative, reviewed in [10, 11]. Moreover resistance to HER2 targeted therapy is now a major clinical dilemma [12]. The high frequency and multiple potential mechanisms of resistance led us to seek the identity of genes that are directly regulated by HER2-stimulated *signal* transduction pathways.

Here, we measured transcription resulting from ectopic HER2 overexpression in a breast cell culture model and compared these data to expression in breast cancer cell lines and breast cancer tissues with and without naturally amplified HER2. In addition, we measured transcriptional potential in cell lines as determined by measuring the binding of RNA Polymerase II (POLII) to genes [13] to define a class of genes that are poised for transcription in HER2 expressing cell lines and are differentially expressed in a HER2-dependent manner. The expression values were compared to those in tumors from humans where the tumor exists within a complete microenvironment. Studies by others have shown the importance of tumor microenvironment in HER2 tumorigenesis [14, 15]. Our studies of HER2-expressing cells reveal that HER2 expression promotes a massive rearrangement of the gene regulation pattern that greatly broadens the biology of HER2, termed the HER2 Regulon. Further, we identified a subset of genes “poised” in HER2 expressing breast cancer cell lines that require the tumor microenvironment for transcriptional regulation. Within this class of genes are pathways known to play roles in stem cells proliferation and self-renewal, such as Hedgehog, Notch and WNT as well as regulatory networks of the node proteins OCT3/4, NANOG, and SOX2. Indeed this class of HER2-dependent and microenvironment-dependent genes commonly contains response elements of transcription factors that medicate OCT3/4, NANOG, and SOX2. These observations support and extend recent evidence that indicates the existence of Cancer Stem Cells (CSCs) in HER2 positive breast cancer with the phenotype of CD44+/CD24−/lin−, and ALDH+ [16]. The results identify a large cohort of genes in the HER2 Regulon whose activity depends on the expression of HER2 and tumor microenvironment.

## RESULTS

### HER2-dependent gene expression in breast cancer cell lines and tumors

We performed whole genome expression analysis on a series of cell lines using U133plus2 arrays with ~54,000 probe sets. We studied MCF7 breast cancer (BCa) cells that in their natural state do not express HER2, and constructed a line, MCF7HER2, that expresses large amounts of active HER2 (Figure S1). We compared these results with expression data from breast cancer cell lines with naturally amplified HER2: BT474 and MDA453. We also compared expression profiles in these cell lines with the measured values for existing profiles of HER2+/− primary breast tumors, totaling 812 primary breast cancer cases in five data sets [17] (Table 1). For this latter comparison the top 35% of tissues with the highest HER2-expression were taken as HER2^+^ and the bottom 35% of tissues with the least HER2 expression were taken as HER2^−^.

**Table 1:** Number of breast cancer cases. Five large expression array data sets from 812 primary breast cancers [17]were normalized and classified as HER2 positive and negative based on HER2 expression levels. The number of cases for each dataset and the total number of cases that are included in this study are shown

Primary tissue datasets	1	2	3	4	5	Total
Number of tumor cases	197	173	115	247	80	812

**Figure 1:**
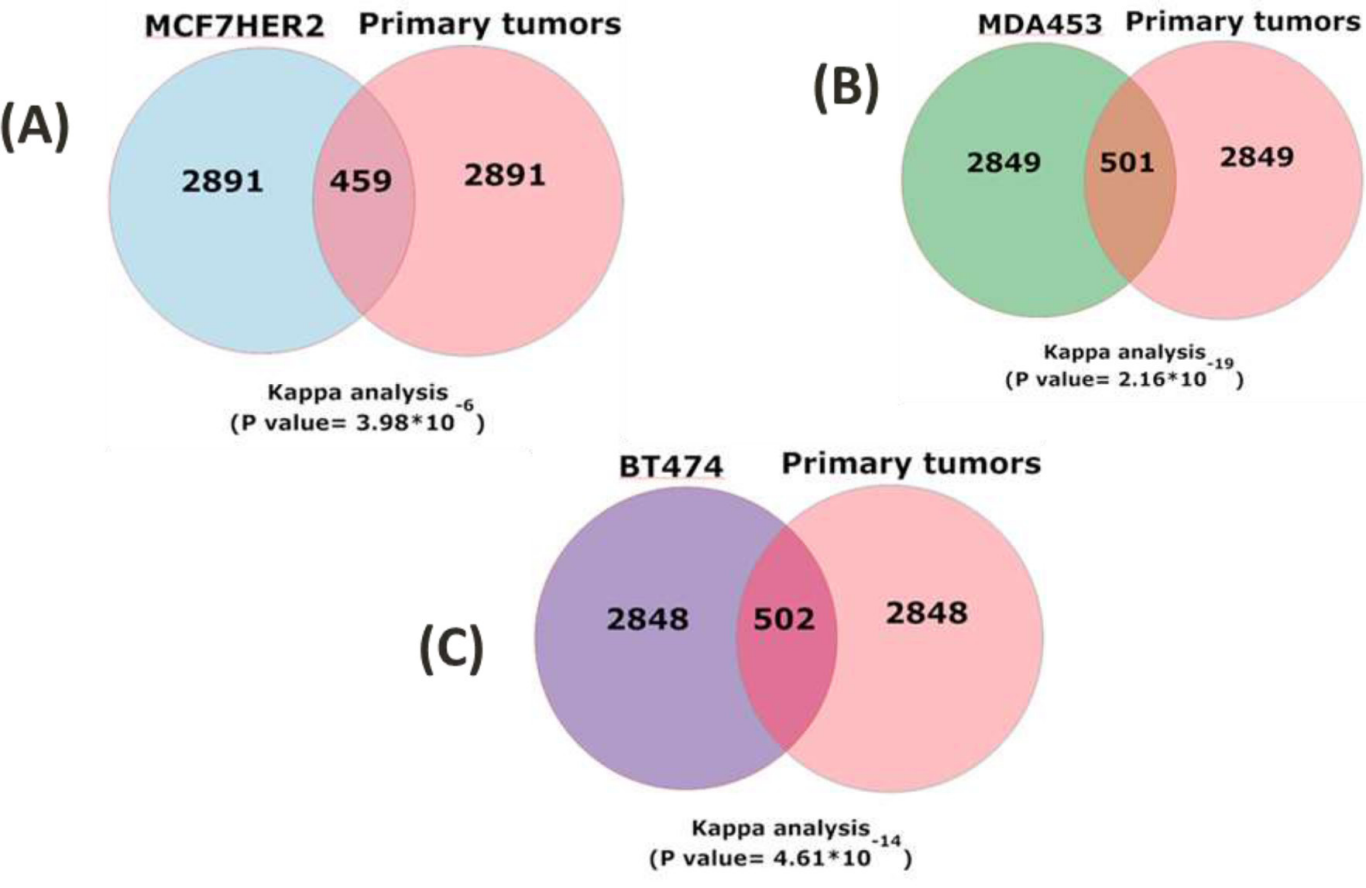
Concordant HER2-correlated changes in gene expression in cell lines and primary breast cancer tissue Expression array data from 812 primary breast cancers were collected, normalized and merged together (20). The 35% highest expressed HER2 samples are considered as HER2 positive and the 35% lowest expressed HER2 are considered as HER2 negative. The gene differential expression analysis was performed on HER2^+^ and HER2^−^ tumor samples by LIMMA. 3350 significant genes with p-value less than 0.05 were selected and compared to 3350 transcripts with the most significant changes in cell lines (p < 0.05). Kappa analysis measured the significance of directionality for (A) MCF7HER2 *vs* primary tumors, (B) MDA453 *vs* primary tumors and (C) BT474 vs primary tumors.

Statistically significant (*p* < 0.05, Materials and Methods) differentially expressed genes in each HER2 expressing cell line *vs.* the non HER2 expressing cell line (top 3350, all *p* < 0.05) were compared to the most significant 3350 (all *p* < 0.05) genes from primary tissue datasets. The overlapping genes between each cell line and the primary tissues were overwhelmingly regulated in the same direction in cell lines and in breast cancer tissues; MCF7HER2, 273/459 (60%); BT474, 335/502 (67%); and MDA453, 349/502 (70%) respectively. Agreement analyses for these comparisons were all significant (Kappa statistics, *p* < 0.0001) (Figure 1, Table 2). The same comparisons were performed on randomly selected genes and kappa values were calculated for 1000 rounds. The kappa values averaged ~0.05, near random expectation.

**Table 2:** Statistical evaluation of comparative gene expression The 3350 transcripts with the most significant changes in cell lines (p < 0.05) were compared to all transcripts (p < 0.05) in the five cancer data sets. Kappa analysis measured the significance of directionality. The number of up and down (↑,↓) regulated genes with the same direction of regulation in each cell lines compared to primary tissues are shown.

Concordant expression with 812 primary breast tumors	MCFHER2	BT474	MDA453
Genes with the same direction of regulation	122↑, 151↓	196↑, 139↓	202↑, 147↓
Number of genes with the opposite direction of regulation	186	167	152
Kappa Statistics	0.201	0.329	0.391
Standard Error	0.045	0.044	0.044
Z score	4.466	7.451	8.929
P value	3.98E-06	4.61E-14	2.16E-19

In order to validate the tissue analysis based on partitioning of the 812 breast cancer tissue using the 35% thresholds to define high and low HER2 expression, we next compared the transcript levels of the 3350 genes that were differentially expressed in our definition of HER2+/− tumor tissues to independent RNA-seq data [18]. The independent data consisted of 8 cases of HER2-positive breast cancer and 24 cases of HER2-negative breast cancers composed of 8 benign breast lesions defined as free of DCIS or invasive cancer, 8 ER+ (estrogen receptor positive) cases, and 8 cases of triple negative breast cancer (TN, estrogen receptor negative, progesterone receptor negative and HER2 negative) [18]. 685 HER2-positive specific genes were identified. When we compared this group of genes with our gene list we identified 169 overlapping genes which is highly statistically significant (*p* < 0.0001) based on resampling simulations carried out 10000 times. These results using independent and validated high HER2-expressing tumors strongly support the analysis based on the two high- and low-HER2 cohorts that we defined using microarray data from 812 breast cancer tissues.

### Genes poised for transcription

Although primary tumors and the three cell lines exhibit overall similar regulation of HER2-dependent genes, about 30% of HER2-correlated genes are regulated in a different direction in the primary tumors *vs.* cell lines. In addition, the majority of genes showing expression correlation with HER2 were unique to particular cell lines or to primary tumors. Therefore, we examined the hypothesis that there are a group of genes in HER2 expressing cell lines that are ready to be expressed but are not expressed. Such non-expression could be due to a lack of signals that would occur in the tumor environment in the patient [19]. For this test, we compared transcription profiles of HER2+/− cell lines to the distribution of RNA Polymerase II (POL II) bound to promoters and the adjacent exons. RNA Polymerase II (POL II) was chosen as a probe for HER2-directed gene regulation because HER2 is not a transcription factor and there is, as yet, no well-defined small number of transcription factors known that mediate gene regulation of the pathways regulated by HER2.

Many details of the mechanism of transcription by POL II are now known through studies of *Drosophila melanogaster, yeast* and *E. coli* [13, 20-22]. Of the three well-recognized RNA polymerases, POL II is the major non-nucleolar polymerase of transcription. Promoter binding occurs in the region of the transcriptional start site (TSS) of protein coding and ncRNA genes, in association with a large complex of initiation factors to form the promoter initiation complex (PIC). POL II may remain poised or stalled in this state. The initiation of transcription involves further association with specific transcription factors and TATA-binding factors, chromatin modification and phosphorylation of the C-terminus of the largest of the 12 POL II subunits. For example elongation is associated with gain of phosphorylation at ser5 and chromatin modifications leading to H3K79me2. A number of variations in regulation are known such as the association of promoter-bound POL II with distant 5′ enhancer elements by DNA looping. POL II may be engaged in limited motion leading to short ~35 nt transcripts or “abortive” transcription and “divergent” transcription along the antisense strand. Within coding sequences where transcript elongation is occurring, further pausing is commonly detected in one or more 3′ sites. It has been shown that POL II is stalled upstream of important transcriptional factors such as c-Myc in both yeast and human embryo stem cells (ESCs), indicating that some POL II locations might constitute nuclear hallmarks important for cell growth and development [23].

### Genes poised for transcription (POLII bound) in a model of HER2 overexpression

Data was obtained using chromatin immunoprecipitation (ChIP) with antibodies to POL II and Agilent promoter arrays that contained multiple probes for ~17,000 genes, with probes concentrated in the promoter and first exons. Unexpectedly, we observed that *de novo* expression of HER2 in MCF7 cells, *i.e.* MCF7HER2 cells, is correlated with POL II interaction with 606 genes (*p* < 0.05) of which only 20% of these new interactions exhibited significant up or down transcription changes (*p* < 0.05). 678 other genes lost POL II binding upon expression of HER2 (Table S1). These observations indicate that HER2 induces a massive rearrangement in POL II binding at promoters. Moreover this picture is an underestimate because Agilent arrays do not sample “long” genes of multiple exons that extend well 3′ of 2500 bp from the TSS. These results therefore represent a very large but still incomplete sampling of the effects of the expression of HER2.

Pathway analysis of genes with POL II binding sites in MCF7HER2 (606 genes) using Database for Annotation, Visualization and Integrated Discovery (DAVID) [24] bioinformatics produced a list of five highly significant gene ontology (GO) terms (Benjamini score 8.20E-07 to 9.5E-04) focused in five main functions, homeobox, developmental, kinase, tyrosine protein kinase and phosphotransferase.

Our data identified more than 30 homeobox genes that gained POL II binding sites in HER2 expressing breast cancer cell line (*e.g.*, MCF7HER2), but not the control MCF7 cells with no HER2 expression. Among POL II bound homeobox genes is HOXB7 which has been reported to promote tumor progression, survival and metastasis once tumorigenesis has begun in HER2 overexpressing breast cancer [25]. It has been shown that POL II stalls at the promoter region of HOXC6 and HOXC8 in mouse embryonic stem cell [26]. Moreover, many of the identified POL II bound homeobox genes here have been shown to be associated with three transcriptional factors NANOG, OCT3/4 and SOX2 in both normal and tumors cells [27]. These transcription factors and their associated genes have the capacity to control the self-renewal and pluripotency of embryonic stem cells. A recent study conducted by Hee Noh, *et al.* has shown that NANOG activates AKT signaling *via* T cell leukemia /protein 1a (Tol 1A) which, in turn, promotes a stem cell-like phenotype and immune evasion in cancer cells [28]. There are numerous reports of association between activated AKT signaling pathway and HER2 overexpression in breast cancer [29]. However, the association between NANOG/AKT and HER2 over expression in breast neoplasia has yet to be fully investigated.

As a control we applied the same functional analysis procedures to the 678 unique genes that were found to bind POL II in MCF7 cells which do not express HER2. The analysis revealed a strikingly different set of functions such as for glycoproteins, transport proteins, cell adhesion proteins, phosphoproteins, and voltage gated channels. Moreover the fit of the 678 genes to these functional groups exhibited markedly higher probabilities, 5 × 10^−3^ – 10^−2^ *vs.* 10^−3^ – 10^−7^. These observations argue that the genes identified for HER2-expressing cells are specific HER2-dependent POL II binding genes.

### Genes poised for transcription (POL II bound) in breast cancer cell lines with acquired amplification of HER2

We extended the ChIP-chip analysis to two human BCa cell lines that exhibit marked amplification of HER2 and very high HER2 protein levels (*e.g.,* Figure S1), BT474 cells and MDA453 cells (Table S1). 266 and 285 of the 606 (POL II bound genes in MCF7HER2 cell line) genes were detected as significantly bound (*p* < 0.05) in MDA453 and BT474 respectively. The overlap among these two groups of genes that bound POL II among the three cell lines is significant (*p* < 0.008) when compared to simulation studies of randomly selected genes from both lines with amplified HER2. The observations indicate the reproducibility of the results based on the MCF7HER2-MCF7pcDNA model and indicate that the model system is relevant to the effects of amplified HER2 in breast cancer.

We quantified the amount and location of POL II binding in each promoter region using previously defined POL II stalling index with slight modification [13]. POL II stalling index was determined for all three high HER2 expressers (MCF7HER2, BT474 and MDA453) in compared to control cells (MCF7pcDNA). Our results illustrate a large effect of HER2 overexpression in shifting the POL II binding site toward the downstream of the TSS as indicated by stalling index (Figure S4).

When compared POL II binding with gene expression most genes had no POL II binding and tended to be among the genes that were not transcribed. Among the HER2-correlated binding events, some genes had strong POL II binding in their promoters (Figure S4) and these genes also tended to be among those that were not transcribed. These promoters are presumably where transcription is poised to occur but is not active [13]. Finally, there were genes that had weak or intermediate binding of POL II; this latter class was more often associated with statistically significant differentially expressed genes (Figure S5).

**Figure 2:**
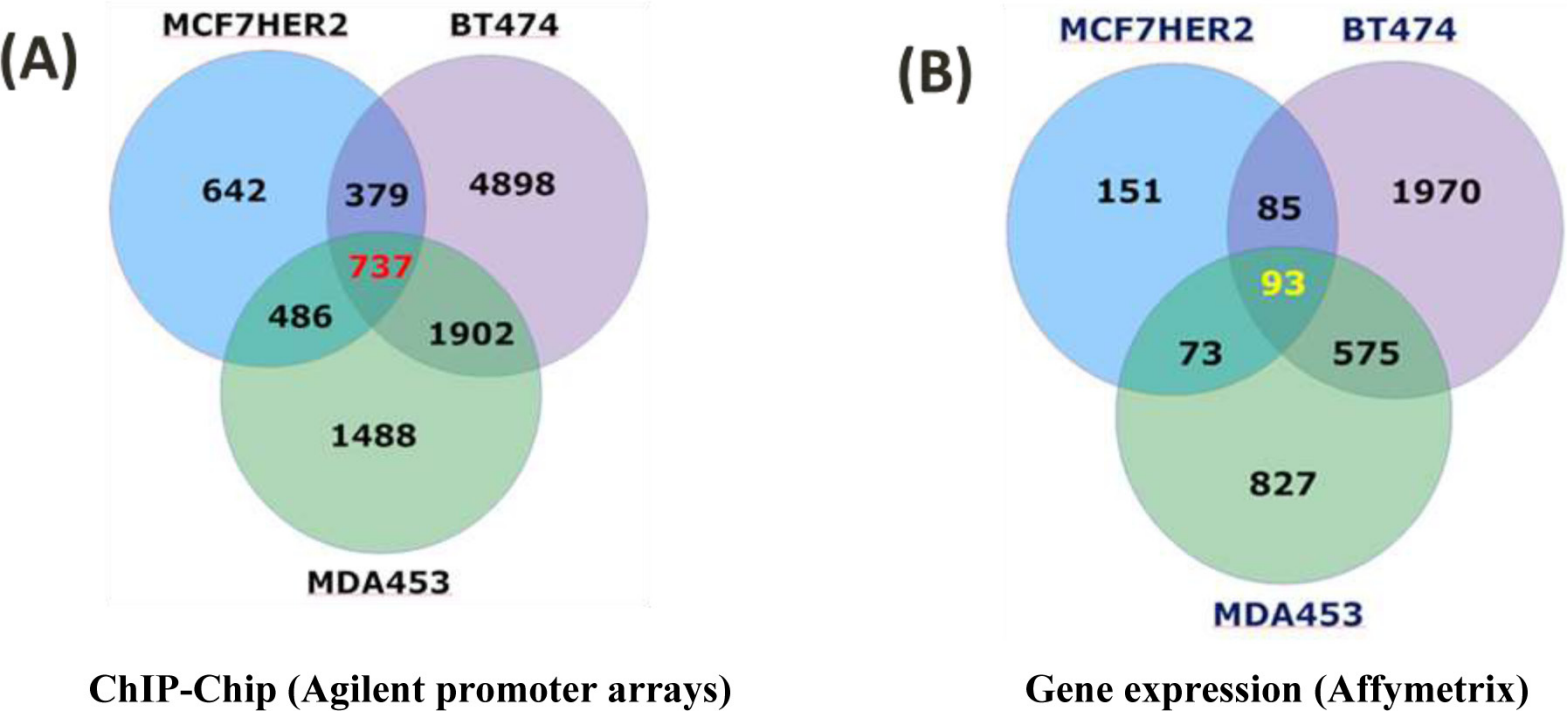
ChIP-chip and Affymetrix gene expression results for HER2-dependent changes in cell lines (A) 737 genes POL II bound in all three cell lines highly expressing HER2, but not in cells without HER2 expression (ChIP-chip). (B) 93 of these genes were also differentially expressed in high HER2 cell lines *vs* cells without HER2 expression (MCF7-pcDNA) (Affymetrix U133 +2). 51 of these 93 genes are regulated in the same direction in all three high HER2 expressing cells. 686 genes have POL II binding sites “poised” and no detectable changes in gene expression in *all* three high HER2 cells. When compared to primary tissue datasets, 113 of these 686 genes were differentially expressed (p < 0.05) in HER2+/− primary tumor tissue.

A group of “relevant” genes (a total of 737) were defined as those with detectable POL II binding (both tight and loose binders with *p* < 0.05 and 0.05 < *p* < 0.13, respectively (Table S1, Figure 2), explained in Supplementary Material and Methods, both in the promoter region and downstream of the transcriptional start sites (TSS) in *all three* cell types that expressed high levels of HER2 (MCF7 HER2, as well as in the naturally high expressing BT474 and MDA453 cell lines), but not in the MCF7 controls that do not express HER2 (Figure 2). These genes are termed the HER2 Regulon here. 93 of these genes were transcriptionally regulated (all with *p* < 0.05) in HER2 expressing breast cancer cell lines when compared to those cells that do not express HER2. 51 of these genes were regulated in the same direction in all three cell lines with high HER2 expression. Of such genes, 36 were down regulated and 15 up regulated. Moreover, 36 additional genes of the 93 genes were found to be regulated in the same direction in two of the three cell line comparisons (Table S2).

Next we asked whether those gene transcripts that are regulated in the same direction (51 genes) in HER2^+^ *versus* HER2^−^ cell lines are in concordance with the expression levels in HER2^+^ *versus* HER2^−^ tissues based on the analysis of the 812 primary tissue datasets. 28 of the 51 genes were assayed among the five external tumor tissue datasets (812 cases). 13 of these 28 genes are significantly differentially expressed in the primary tumor datasets (all with *p* < 0.05) and 10 of the 13 genes are transcriptionally regulated in the same direction in both high HER2 cell lines and the primary tissues (Kappa value, 0.54 and *p* < 0.02) (Table S2).

### Up and down-regulated transcripts in high HER2 expressing cell lines from POLII bound genes

Among down-regulated genes are; MRAS, SOCS5, GAB2, STMN3, PPP3CC. Five genes (SEMA3F, BLVRB, PTPRF, MARCKS, and CQQ6) are up regulated both in HER2 positive cell lines and in high HER2 expressing primary breast tumor tissues (Table S2). Among the discrepant genes between cell lines and primary breast tumors; one gene (CDKN2D, cyclin dependent inhibitor 2D, inhibits CDK4) is up regulated in three high HER2 expressing breast cancer cell lines and down regulated in HER2^+^ expressing breast carcinomas whereas, two other genes (CNOT2, PAPSS2) are down regulated in HER2 expressing cell lines and up regulated in HER2 overexpressing breast cancer tissues. CNOT2 (CCR4 associated factor 2) regulates mRNA synthesis through interaction with HDAC1 and is a regulator of stem cell maintenance [30]. This gene binds to and inhibits TFIID which binds to the core promoter to position POL II properly and acts as a channel for regulatory signals. 23 of the 51 genes that are regulated in the cell lines in an HER2 dependent manner were not assayed in the combined external breast tumor tissue datasets however, all are transcriptionally regulated in the same direction among all three high HER2 expressing cancer cell lines (*p* < 0.05) (Table S2). Several of these genes are described in breast cancer including FN1, Fibronectin1, which is down-regulated in high HER2 expressing cell lines, and has been reported to be suppressed in metastatic breast cancer [31].

### POL II bound genes in high HER2 expressing cell lines that are not transcribed

Our data revealed the identity of 737 genes with POL II binding sites in HER2^+^ cells. 686 of such genes are not transcriptionally regulated in the same direction in *all three* HER2+/− comparisons (Figure 2). These are POL II bound genes “poised” in HER2 expressing cell lines without transcripts that are differentially regulated in HER2 dependent manner. We compared the expression levels of 686 POL II bound genes with no significant differential expression in *all* HER2+/− cell lines to the 3350 significantly HER2 dependent differentially expressed genes in five primary tissue data sets totaling of 812 cases. 113 genes were significantly *differentially* expressed in HER2^+^ primary tissues compared to HER2^−^ primary tissues (Table 3). We speculate this is due to the dramatically different context of cells in culture *versus* in the whole tumor. Of 113 such class of genes, 65 are up regulated and 48 are down regulated in HER2^+^ *vs* HER2^−^ primary breast cancer. Among up regulated genes are SDC1[32], DUSP6 [33], VASP [34], IDH2 [35], DDR1 [36], GPC1 [37], SQSTM1 [38], and among down regulated genes are RHEB [39], IRS-2 [40], HSPB2 [41] and RAP1A [42]. Several of these genes have been reported previously to be associated with high levels of HER2 expression in human breast and ovarian neoplasia (Table 3).

**Table 3:** 113 genes with HER2-dependent POL II binding but no expression in cell lines and significant differential expression between high and low HER2-expressing breast cancer tissues (p < 0.05)

Gene Name	HER2+/−Primary tissue LogFC	P– Value	Gene Name	HER2+/− Primary tissue LogFC	P– Value
SDC1	0.73	2.98E-18	IGF2R	0.19	0.0236
C7orf24	0.71	2.13E-17	SPECC1L	0.19	0.0247
CTDSP1	0.53	2.25E-10	ALG3	0.19	0.0255
CISH	0.53	3.00E-10	NUMA1	0.17	0.0383
DUSP6	0.53	4.07E-10	WDR33	0.17	0.0399
SRPK3	0.49	5.78E-09	SEC24B	0.17	0.0405
NDUFA3	0.48	1.02E-08	TOR3A	0.17	0.0406
DDR1	0.46	4.10E-08	SQSTM1	0.17	0.0422
PPOX	0.43	3.04E-07	FABP4	−0.17	0.05
TACSTD2	0.42	4.52E-07	TM4SF1	−0.17	0.048
NCSTN	0.42	6.70E-07	ADCY1	−0.17	0.047
VASP	0.42	7.04E-07	USP2	−0.17	0.046
SLC39A1	0.41	1.33E-06	KIAA0999	−0.17	0.042
FGFR1OP	0.37	8.37E-06	STK24	−0.17	0.041
SGMS1	0.37	1.13E-05	EPHA4	−0.17	0.04
CELSR3	0.35	3.15E-05	BST1	−0.18	0.033
LAD1	0.35	3.64E-05	RIF1	−0.19	0.026
SEPW1	0.35	4.06E-05	R3HDM1	−0.19	0.023
FRAG1	0.34	5.09E-05	RHOBTB3	−0.19	0.021
GPC1	0.33	7.12E-05	TLX1	−0.2	0.019
GOLGB1	0.32	0.0001	CCNL1	−0.2	0.019
XKR8	0.32	0.0001	PDE2A	−0.2	0.016
KCNK1	0.32	0.0001	SPIB	−0.21	0.014
PFDN2	0.32	0.0002	RPL10A	−0.21	0.013
ACOX2	0.32	0.0002	KCNAB2	−0.21	0.013
PRKCZ	0.32	0.0002	ITGAE	−0.21	0.013
DKK1	0.31	0.0002	RBBP7	−0.21	0.011
MARK2	0.31	0.0003	NUS1	−0.22	0.009
ATP5G1	0.3	0.0003	CLIC4	−0.22	0.008
IDH2	0.3	0.0003	CSNK2A2	−0.22	0.008
XRCC5	0.29	0.0005	CHL1	−0.24	0.005
ACAT2	0.29	0.0005	NEFH	−0.24	0.004
GATA3	0.28	0.001	RAP1A	−0.25	0.003
BRD2	0.27	0.0011	CBS	−0.25	0.003
TES	0.27	0.0015	FBXO2	−0.25	0.002
GCNT1	0.26	0.0017	PQLC1	−0.26	0.002
LRRC23	0.26	0.0018	PTCH1	−0.26	0.002
ZNHIT2	0.26	0.0022	WDR77	−0.27	0.002
TMEM115	0.25	0.0025	THOC5	−0.27	0.001
FNBP1L	0.25	0.0025	TCP11L1	−0.28	0.001
STK16	0.25	0.003	DYNLT3	−0.29	0.001
CTBP2	0.25	0.003	FGL2	−0.29	0
ADAMTS13	0.25	0.0031	TUBGCP3	−0.3	0
AP2S1	0.25	0.0035	OTOF	−0.3	0
KIAA0195	0.24	0.004	HSPB2	−0.31	0
SNAPC5	0.24	0.0043	VRK2	−0.35	0
CNN2	0.23	0.006	RHEB	−0.35	0
BBS1	0.23	0.0074	RTP4	−0.37	0
BCAR3	0.22	0.0094	GABRP	−0.38	0
RNF141	0.22	0.0098	VAMP3	−0.39	0
TINF2	0.2	0.0154	CAPN6	−0.4	0
TETRAN	0.2	0.0181	ANKRD15	−0.41	0
MMP15	0.2	0.0191	STAC	−0.45	0
WHSC1	0.19	0.0215	IRS2	−0.47	0
PMPCA	0.19	0.0216	EPB41L2	−0.49	0
HNRPDL	0.19	0.0223	CD320	−0.58	0

### Pathway analysis of genes poised for transcription in cell lines and differentially transcribed in breast cancer

Functional relationships of the 113 differentially expressed genes were examined by computer-assisted searches using MetaCore software and Strand –NGS pathway analysis tools (Agilent). Two main processes were overrepresented in this subclass of HER2 regulated genes including inflammation, immune response especially for interleukin 5, 9, 4, 1,13 and developmental pathways such as, Hedgehog, Notch and Wnt. Previous studies using gene expression analysis of different breast cancer cell types have indicated that inflammation within the tumor microenvironment (cellular context) of breast tumors may enhance tumor progression through increasing motility and invasion [43]. The reciprocal interactions between tumor and stromal cells through cytokines signaling, especially IL6 and IL8, mediate tumor progression, metastasis and resistance to therapy ( reviewed in [44]). Korkaya *et al* have shown that the activation of an IL6 inflammatory loop mediates Trastuzumab resistance in HER2^+^ breast cancer by expanding a cancer stem cell population [14]. Among signal transduction pathways associated with HER2 regulated “poised” class of 113 tissue dependent genes were insulin, androgen receptor signaling cross talk, Hedgehog, Notch and Wnt signaling. These findings are consistent with published data that implicates the cross-talk among HER2, Notch, Hedgehog and Wnt pathways in HER2 positive breast cancers [45]. High HER2 expressing tumor cells display activated Notch signaling [45]. Both HER2 and Notch signaling play roles in regulating cancer stem cell [45].

**Figure 3:**
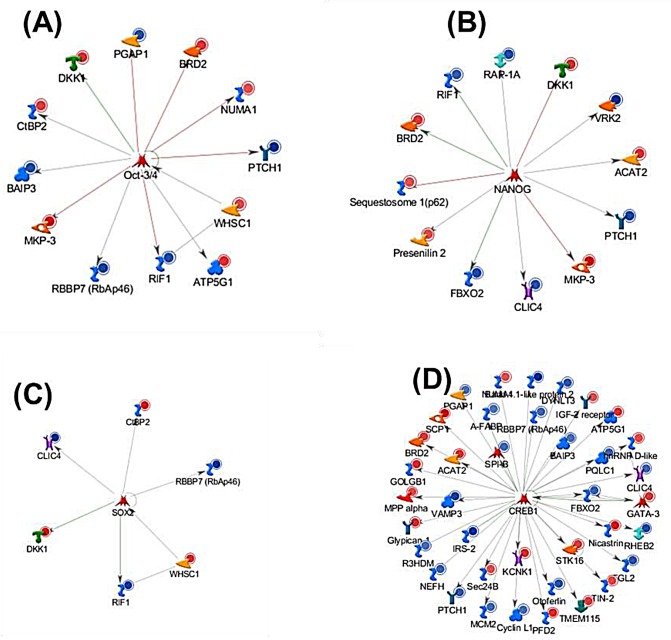
Gene ontology (MetaCore) analysis of 113 genes with poised POL II binding sites in high HER2-expressing breast cancer cell lines and differentially expressed in HER2+/− breast carcinomas Networks are graphically visualized as nodes (proteins) and edges as relationship between proteins. The line colors designate the nature of the interaction; red= negative effects, green=positive effects gray is unspecified. Blue and red circles represent down regulated and up regulated genes in HER2+/− primary breast tissues respectively. Transcriptional factor (A) OCT3/4, (B) NANOG, (C) SOX2 and (D) CREB1 interact with 11, 11, 6 and 38 genes respectively.

Additionally, many of the 113 HER2 regulated genes were associated with stem cell and progenitor cell control, as indicated by networks centered on NANOG (FBXO2, CLIC4, PTCH1, RIF1, VRK2, BRD2, Presenilin 2, RAP-1A, Sequestosome 1(p62), OCT3/4 (ATP5G1, BAIP3, BRD2, CtBP2, NUMA1, PGAP1, PTCH1, RBBP7(RbAp46), RIF1, WHSC1) and SOX2 (CtBP2, CLIC4, DKK1) (Figure 3). Previous studies have demonstrated that core transcription factors, such as *NANOG, SOX2* and*, OCT3/4* are involved in the maintenance of pluripotency and self-renewal in embryonic stem cells (ESCs), and have been identified in tumors of various origins (reviewed in [25]). Indeed we have confirmed significantly increased expression of NANOG, SOX2 and, OCT3/4 in cultures of “mammospheres” of MCF7HER2 cells compared to attached cultures and to MCF7pcDNA3 cells (Figure S8). Thus, a role for stem cells in proliferation of HER2-regulated breast cancer is highly suggested.

38 of the 113 genes were associated with CREB1 (cAMP responsive elements binding protein) which regulate aromatase in breast cancer. It has been reported that over-expression of aromatase in adipose tissue surrounding breast tumor (microenvironment) could arise through increase in both CREB expression and CREB transcriptional activity [46] (Figure 3). Moreover, expression of CREB1 has been reported to be associated with poor prognosis and metastatic breast cancer [47]. In all the four node genes, NANOG, SOX2, OCT3/4, and CREB1, are associated with the regulation of 57 of the 113 genes (Figure 3). The gene regulation changes that are tissue context-dependent represent a fundamental new class for understanding HER2 mechanisms in breast cancer.

487 more genes with POL II binding in HER2 positive cell lines were identified. This class of genes has no transcripts that were differentially expressed in HER2^+^/^−^ breast cancer cell lines. These genes were not assayed in the five combined breast cancer tissue datasets (i.e. 3350 significant genes in the merged primary data). A literature search using the MetaCore pathway analysis tool revealed an association of 124 of these 487 genes with breast neoplasia. The remaining genes have no previously documented association.

### Transcription factors and their DNA binding sites

We sought to determine whether the response element sequences (RESs) of the 113 tissue context dependent genes indicated regulation by transcription factors consistent with the regulatory networks identified here. We utilized TRANSFAC (TRANScription FACtor database) [48-50]. TRANSFAC analysis identified that 99% of the 113 genes (112/113 of the tissue context dependent genes) contain one or more putative RESs with the average number of sites per gene of 39.37. Match^TM^ revealed the identity of 122 Transcriptional factors whose binding sites were highly enriched in the promoter regions of (+ 500 bp around TSS) of 113 *tissue context dependent genes.* Among these overrepresented transcription factors are NANOG, CREB1, CPBP, BEN, PREB-1, SOX10, POU2F1 (OCT1) and POU6F1 (POU domain, Class 6, Transcriptional factor 1) (Table 4 and Figure S7). Many of the transcription factors utilize Homeobox or HOX RESs. One or more HOX RESs were found in the promoter region of 16 of the 113 tissue context dependent genes. Several of the TRANASFAC-identified transcription factors are the same (e.g. NANOG, CREB1, CPBP) or closely related (*e.g.,* SOX10, OCT1) as the genes identified by pathway analysis of the 113 tissue context-dependent genes above and in Figure 3. Other TRANSFAC-identified factors are not members of the node gene networks of Figure 3 but known mediators of network members. For example, at least ten of the TRNASFAC-identified transcription factors medicate the nodal gene CREB1 (Figure 3 and Figure S7). The common enrichment of RESs in the 113 genes that correspond to or medicate the nodal genes and their network members further supports the regulatory networks based in NANOG, SOX2, OCT3/4, and CREB1 of Figure 3 deduced here.

**Table 4:**
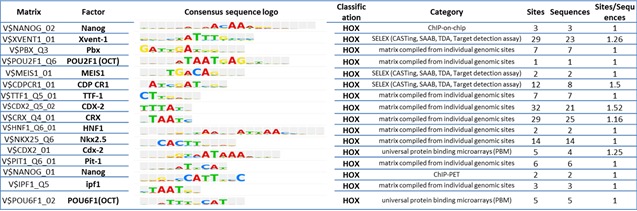
Homeobox enriched Regulatory Element Sequences in the promoter regions of tissue context dependent genes We used the Match ^TM^ algorithm and TRANSFAC matrices to predict regulatory element sequences within the promoter regions (+ 500 bp around the TSS) of 113 *tissue context dependent* genes. The binding factors, the nucleotide position frequency and sequence logo, and the source of extracted data (category column) such as ChIP-on-, SELEX or matrix compiled from individual genomic sites are shown.

## DISCUSSION

Breast cancer, including HER2 positive, starts years before current detection which is often self-reported. Huge efforts have been spent on early detection and treatment. New agents targeting HER2 have been developed and have greatly improved treatment choices, response rates and length of remission. However, the general pattern remains that HER2 positive breast cancer subsequently progresses. Extensive research has shed light on numerous potential mechanisms that account for its devastating effects in breast cancer. Gene amplification over a large region flanking the HER2 gene leads to many copies of the gene and high expression of up to ~400,000 receptors molecules per cell located on the cell surface. The receptor readily dimerizes leading to autophosphorylation and activation. The activated receptor in turn acts on at least two crucial pathways. The Ras/Raf/Map kinase/Erk kinase pathways leading to the activation of genes that promote cell proliferation. A second pathway is mediated by the P13K/Akt and promotes down regulation of several intermediates of apoptosis thereby promoting increased cell survival. Together these complementary effects provide an attractive rationale for the oncogenic role of HER2. Recently a potential example of how other pathways may be involved in HER2 signaling has come to light in the form of an alternatively spliced HER2 transcript missing exon 16 leading to an altered form of HER2 (HER2Δ16) significantly associated with locally disseminated metastatic breast cancer [51]. As another example an amino truncation of HER2, p95HER2, has been detected recently and is associated with an aggressive phenotype including nodal metastasis in human breast cancer [52]. Whether any of these mechanisms utilize a common function(s) *via* coordinated gene regulation is not known.

We examined the hypothesis that there exist unknown HER2-stimulated signal transduction pathways leading to coordinated gene regulation that has not previously been recognized. High throughput techniques such as expression microarray analyses have generated invaluable information about the differential gene expression patterns in HER2^+^ and HER2^−^ breast cancer cell lines [53]. Here, high throughput ChIP-chip analyses and comparison of cell lines to breast cancer tumors revealed fundamental new knowledge about HER2. This is the first insight that HER2-expressing breast cancer cell lines exhibit the altered regulation of *hundreds* of genes termed here the HER2 Regulon.

We revealed an interesting class of 113 POL II bound genes “poised” without transcripts that are differentially expressed in a HER2-dependent manner in cell lines and were differentially expressed in HER2+/− breast cancer tissues where both tumor cells and host tumor microenvironment exist (Table 3). Clearly the context of tumor cells *in situ* influences actual HER2-dependent expression of the genes of the HER2 Regulon.

Pathway analysis of 113 differentially expressed genes identified enrichment in GO terms for cell adhesion, regulation of translational initiation, inflammation, immune response especially for Interleukins 5, 9,1,4 and 13, cytoskeleton remodeling, membrane domain ectodomain proteolysis (the proteolytic cleavage of trans membrane proteins and release of their extracellular domain), and development through Wnt, Hedgehog and Notch signal transduction pathways. A large group of genes are associated with stem cell and progenitor cell control as indicated by networks centered on NANOG and OCT3/4 and SOX2. These genes may play roles in regulation of cancer stem like cells or pluripotent cells capable of self-renewal as well as mitogenic expansion of the tumor in HER2-postive breast cancer and may be key to the growth and survival of metastatic HER2 positive breast cancer. This observation is in agreement with recent studies that activation of Notch, Wnt and Hedgehog signaling pathways in CSC require both tumor epithelial and tumor associate stromal cell (*e.g.* tumor microenvironment) [15, 16]. Thus, the role of stem cells proliferation in HER2 regulated breast cancer that is dependent on interactions with the microenvironment in the regulation of a subset of HER2 Regulon genes is highly suggested.

Moreover, we investigated the role of transcriptional factors and their regulatory element sequences in HER2 positive breast cancer using the set of 113 genes that are poised in HER2^+^ cell lines but required breast tumor tissue microenvironment for expression (*i.e.*, *issue-context dependent*) using BIOBASE databases tools. Predicted regulatory element sequences for NANOG, CREB1, CPBP, BEN, PREB-1, SOX10, and POU2F1 (OCT1) POU6F1 and MEIS1 were highly enriched in the promoter regions of the tissue context dependent genes suggesting their possible roles in stem cells proliferation in HER2-regulated breast cancer tissue.

In summary, the findings from this study define HER2-dependent POL II binding to genes in cell lines and breast cancer tissues, the HER2 Regulon, and a subset that exhibit tissue context dependent expression. These findings have the potential to transform our views of how HER2 works. The findings appear relevant to CSC biology including genes of the Notch WNT, Hedgehog, NANOG, OCT43/4 and SOX2 families, immune response, growth, cell adhesion and others. A fruitful avenue for future study is to dissect genes related to Trastuzumab treatment and mechanism of resistance. Of particular interest is to identifying OCT3/4, NANOG and SOX2, associated genes that exhibit HER2-dependent POL II binding “poised” and are not blocked by Trastuzumab.

## MATERIALS AND METHODS

### Cell lines and transfection

Cell lines expressing pcDNA3, pcDNA3-HER2 were cultured and characterized using methods previously described [51]. Briefly, MCF7 cells engineered to express HER2 or contain the empty vector pcDNA were maintained in MEM supplemented with essential and non-essential amino acids (Invitrogen), Pyruvic acid, 10% glutamine and 10% FBS. Cells were harvested after they reached 80% confluence. Breast cancer cell lines BT474 and MDA453, which were originally derived from HER2-positive breast cancers that exhibited amplified HER2 [54, 55] were purchased from ATCC and cultured using the recommended protocols.

### Protein Expression Analysis, Western blotting

Breast cancer cell lines were lysed and loaded onto NuPAGE® gels (Invitrogen). After electrophoresis was completed, proteins were transferred onto a pre-wet nitrocellulose membrane (Life Technologies). Membranes were rinsed with water twice, and then were blocked 1 hour at room temperature with gentle agitation in freshly prepared Tris buffered saline (TBS) and 1% Tween 20 (TBST) containing either 2% milk or 2% BSA. To enrich phosphopeptides in breast cancer cell lines we utilized phosphopeptides enrichment columns (Clontech). Membranes were incubated with antibodies against HER2, AKT, pAKT, pHER3, pHER2/ErbB2 (Tyr1221/1222), NFKB (Cell Signaling) and HOXA10 (Santa Cruz) diluted in TBST overnight at 4^0^C with agitation. After washing the membranes three times with TBST, they were incubated with the appropriate secondary antibody (Anti Rabbit or Anti Goat IgG) and incubated for 1 hour at room temperature and detected using chemiluminescence reagents (Amersham, ECL Western Blotting detection reagents). Western blot images were quantified using Alpha Ease FC^TM^ (Alpha Innotech Corporation) imager software (Figure S1).

### Chromatin Immunoprecipitation assay (ChIP)

RNA polymerase II (POL II)-bound DNA was captured by a chromatin immunoprecipitation procedure. In order to detect POL II enrichment in target gene promoters, the four breast cancer cell lines were grown at a density of 5 × 10^6^ and ChIP DNA was prepared according to the recommended manufacture protocol (SA biosciences). Briefly, POL II cross linked to DNA *in situ* by addition of 1% formaldehyde to living cells. The antibody to POL II (SA biosciences) was then applied to sonicated chromatin that has been exposed to formaldehyde. Protein A Beads (Champion Chip^TM^ one day kit) was used to precipitate the antibody bound promoter complexes. We purified the captured DNA after reversing cross links by heating to 95^o^C.

Subsequently, Whole Genome Amplification (WGA, Sigma) was utilized to amplify the whole genomic DNA. We analyzed the specific enrichment of ChIP-captured DNA by traditional agarose based gel for the GAPDH and ERCC1 (known POL II target genes) and for an ORF-free region as a negative control (Figure S2A). The immuno-precipitated DNA (IP) and the input DNA were then labeled with florescence labeling dyes Alexa 647 and Alexa 555 respectively, mixed in equal amounts (5μg) and hybridized to a “promoter array” using Agilent human proximal promoter microarray slides (Human promoter, 2 designs-244K, Agilent Technologies).

### Agilent Human Promoter Array and data analysis

We utilized Agilent promoter arrays which probe ~ 17,000 genes with probes (500K probe sets) spaced approximately ~200 nucleotides (nts) apart, and cover −5000 to +2500 nts about the transcriptional start site (TSS). Anti-POL II Chromatin immuno-precipitated DNA was hybridized with an equal amount of genomic DNA (competitive hybridization). Chromatin immunoprecipitation of POL II-bound DNA was carried out with mid-log phase cells (80% confluence), and applied to the Agilent promoter arrays. Significant binding was defined by the Agilent protocol that examined the intensity of the bound DNA signal for each probe sets in comparison to each upstream and downstream neighboring probe set to identify discrete binding sites. The hybridization signal was recorded and the statistically significant data (*p* < 0.05) were analyzed to calculate the enrichment value of immuno-precipitated DNA (IP) *vs.* the whole Cell Extract (WCE). Microarray data were collected for engineered MCF7 system (MCF7HER2, MCF7pcDNA (control) and breast cancer cell lines BT474 and MDA453. All data sets were processed using Agilent Feature Extraction software (version 9.1) and normalized using a linear per array algorithm according to the manufactures protocols for ChIP-chip analysis. DNA analytic software (ChIP module, Agilent) was utilized to determine bound regions in the dataset by using the Whitehead Per-array neighborhood model (Figure S2B). In the neighborhood model all sets of three adjacent binding sites within 1100 bases are examined. The program considers the central probe set “bound” if the *p* value of the composite error-corrected ratio of all three probes (*p_*x_bar) is less than 0.05 (selected *p* value cut off), and if either of the following is true: (1) The *p* values for the central probe set and at least one of its neighbors are less than 0.05 or (2) The *p* value of one of the neighbors of the central probe is less than 0.1. Thus the interaction of POLII and a single DNA binding site, we used the individual probability, *p_*x_bar, as described by Agilent Inc. When more than one site were observed “bound” within −5000/+2500 bp of a given gene’s transcriptional start site (*i.e.* the zone probed by the Agilent array), we derived joint *p* values, p_jt_, and the geometric average probability, p_gav_, determined as follows. For p_jt_ we transformed the product of *p_*x_bar’s, π_n_(*p* x_bar), to a chi-square statistic with degree freedom of 2*n, using Fisher’s method [56], where n is the number of binding sites that are involved in this calculation. The chi-square statistic compared to the null chi-square distribution with mean = 2*n to yield p_JT_ [57]. The modified probability, the geometric average probability, p_gav_, facilitates the comparison of binding strengths of genes or DNA sequences with single binding sites to those with multiple significant POL II binding sites. p_gav_ is derived similar to p_JT_ but using ^n^√ (π_n_(*p_*x_bar), the geometric average, followed by the Chi-square transformation with degree of freedom of 2. The extent of binding of POL II for MCF7HER2 was validated for a randomly selected POL II binding sites (8 sites) using traditional and quantitative ChIP-PCR (Figure S3).

### Statistical analysis of the distribution of POL II binding

The distribution of POL II binding within a gene was determined following a modified procedure for calculation of the Stalling Index (SI) of Young and coworkers, explained further in supplementary materials and methods [13], Figure S4 and Table S1.

### Microarray expression analysis

Total RNA was purified from the two replicate MCF7 engineered cell system and the two naturally expressing HER2 breast cancer cell lines using the Uneasy plus kit (Qiagen). Total RNA samples were processed for hybridization to Affymetrix U133 plus2 Gene Chips. Prior to micro array analysis the integrity of RNAs was assessed using the RNA 6000 Nan lab Chip Kit using Agilent 2100 Bio analyzer. Expression analysis for all samples was assessed using U133 plus2 platform with approximately 54000 probe sets covering 21K annotated genes. Data was normalized using the function “normalize Quintiles” of LIMMA (Linear Model for Microarray Data) Data sets. The LIMMA package from Bio conductor was used to detect differentially expressed genes in HER2 expressing *vs.* non HER2 expressing breast cancer cells. All microarray cDNA expression analysis was performed at the UCI microarray core Facility; (http://dmaf.biochem.uci.edu).

### RNA isolation and quantitative Real time RT-PCR for validation of Affymetrix gene expression array (U133 plus 2)

A total of 53 genes were selected for validation of gene expression results for the MCF7 system as well as the two human breast cancer cell lines BT474and MDA453 using the same RNA source that was utilized for microarray expression analysis. The Total RNA was reversed transcribed in the presence of qScript^tm^ cDNA Super Mix (Quanta Biosciences). The cDNA then was used for expression analysis of the selected genes. Primer sets for the 53 genes were designed using Integrated DNA Technology primer design software and were confirmed by “blasting” each primer sequence against the human genome using NCBI and UCSC Genome Browser. All primer sets for the 53 genes were examined in parallel with duplicates using an ABI7900 sequencer in 384-multiwell format by the SYBR Green method. Primers were found gene specific if corresponding melting curves produced a single sharp peak and the expected amplicon size was observed using agarose electrophoresis. All Cycle Threshold C_t_ values of the genes were normalized to housekeeping gene, *i.e.*, GAPDH. Briefly, for each sample (high HER2 and null HER2 expressing cells) we calculated the difference between the Ct values (ΔCt) for the gene of interest and the house keeping gene GAPDH. The differences in ΔCt ( cycle threshold) values for the gene of interest for each pairwise samples to be compared were calculated as ΔΔCt. The fold changes in gene expression between the high and low HER2 expressing cells was calculated as 2 ^(−ΔΔCt).^ The primer pair sequences and PCR conditions will be provided upon request. For validation, we compared the observed Affymetrix values to those of qPCR by a quantitative correlation analysis. (Table S3 and Figure S6).

### Biological functions and pathway analysis

We utilized commercially available system biology tool MetaCore from Gene GO (Thomson Reuters Inc.) and Strand-NGS (Agilent) pathway analysis tool to further define the significant biological pathways, processes and networks that existed in our gene list. The software (http://www.genego.com) is based on a manually curated database of known molecular interactions, pathways and processes. First, we used multiple experiment analysis for enrichment analysis of data corresponding to gained and lost POL II genes in HER2 expressing cells, by filtering the genes for breast tissue specificity and then mapping them onto selected MetaCore Ontologies such as GeneGO pathway maps, GeneGO process, and GeneGO network. The *p* value throughout MetaCore for maps, processes, and networks are calculated based on the hyper-geometric distributions where the *p* value that essentially represents the probability of any particular mapping arising by chance, given the number of genes in the set of all genes on maps, networks, and processes and genes in our experiments.

### Primary tumors datasets

Five data sets were collected from publically available sources as described [17]. The genes of each data set were first normalized to follow a normal distribution with mean 0 and standard deviation 1. Then the normalized five data sets were merged together for a total of 812 patient samples and 7327 common genes. It is important to note that the microarray platforms used in these studies are completely different. The definitions of HER2 positive and negative patients are based on the gene expression intensity of HER2 in the merged data set. The 35% highest expressed HER2 samples are considered as HER2 positive and the 35% lowest expressed HER2 are considered as HER2 negative. The gene differential expression analysis was performed on HER2 positive and negative samples by LIMMA, identifying a total of 3350 significant genes with *p*-value less than 0.05. To compare our cell line data with the primary tissue data, we used the 3350 genes with the statistically significant expression levels in HER2 expressing cells *vs*. non HER2 control cells. For example, when differentially expressed genes in MDA453 were compared to 3350 significant genes in the merged primary data set, we identified 501 concordant genes. Furthermore, according to the up and down gene regulation status, we constructed a contingency table between our data and merged primary tissue data (Table 1 and 2).

### Primary tumor data set, RNA Seq

Details of the HER2+/− breast cancer RNA-Seq data have been published previously [18]. Mean gene expression was compared between tumors, and 685 genes were differentially expressed in HER2^+^ cases at p < 0.05 when compared to each of the other tumor subsets. We used the HER2 related genes (685) and compared those with our list of 3350 differentially expressed genes in HER+/− tumor tissues.

### Transcription factor RESs of 113 “poised” tissue-context dependent genes

We used a professional version of TRANSFAC (BIOBASE, Qiagen) software to identify transcriptional factors and RESs in the promote region (+500 bp around the TSS) of 113 genes with poised POL II binding sites in high HER2-expressing breast cancer cell lines and differentially expressed in HER2+/− breast cancer tissues *( e.g. tissue context dependen*t) [49, 50]. Table 4 shows a partial list of transcription factor response element sequences and their associated transcriptional factors. We used the Match ^TM^ algorithm and TRANSFAC matrices to predict regulatory element sequences within the promoter. The binding factors, the nucleotide position frequency and sequence logo, and the source of extracted data ( category column) such as ChIP-on-chip, SELEX or matrix compiled from individual genomic sites are shown in Table 4.

The Match^TM^ algorithm uses two scores: 1) the matrix similarity score (MSS) and 2) the core similarity score ( CSS). These scores measure the quality of match between the sequence and a consensus reference sequence which ranges from 0.0 to 1, where 1 denotes the exact match. We used a recommended cut off (predefined by the Match^TM^) to minimize false positive (over prediction) and false negative (under prediction) matches and minimizes the sum of both errors.

### Simulation analyses

Simulations were carried out in R. For analysis of POL II binding genes in cell lines, random sets of human genes from the Agilent array were selected. These sets were the same size as the number of immuno-precipitated genes from BT474 and MDA453. The frequency of overlap of genes in the random list with these 606 genes in the Chip results for MCF7HER2 was determined, and the analysis repeated 10000 times.

The analysis of overlapping genes between the breast cancer tissue arrays [17] and the breast cancer tissue RNA-Seq data [18], proceeded as follows. There were 7326 genes in the first set, of which 3350 were associated with HER2 expression. There were 22323 genes in the second set of which 635 were reported differentially expressed in the RNA-Seq analysis. 161 genes overlapped in both lists. 3350 genes were randomly selected from 7326 genes and 685 genes were randomly selected from the 22323 genes, the overlap was determined and repeated 10000 times. The probability of the observed overlap as chance was estimated as the ratio of the average frequency of random overlap to the observed overlap. The method includes the candidate genes in the random draw and slightly over-estimates the random frequency.

### Mammosphere formation

Details of sphere formation by cancer cell lines were described previously [58]. Briefly, the HER2+/− breast cancer cell lines were grown in MEM medium supplied with 10% FBS, the sub-cultures were depleted from the serum-containing medium and 2-5 × 10^6^ cells were cultured on ultra-low adherent plate (Corning, MA) in MEM supplemented with epidermal growth factor (EGF, 20 ng/ml), basic fibroblast growth factor (FGF, 10 ng/ml), and 1–5% B27 supplements (Life Technologies, CA). The formed spheres were enzymatically dissected into single cell suspension and further subjected to at least two cycles of sphere formation. Total RNA was reversed transcribed using a high capacity cDNA reverse transcription kit (Applied Biosystems). The cDNA was then used for expression analysis of ALDH1, SOX2, NANOG and OCT3/4 employing quantitative real time PCR (Power SYBR Green PCR Master Mix, Applied biosystems). Primer sets for targeting these genes were designed using primer design software from Integrated DNA Technology and were confirmed by “BLASTing” each primer sequence against the human genome using NCBI and UCSC Genome Browser (Table S4). All Cycle Threshold C_t_ values of the genes were normalized to housekeeping gene, *i.e*., GAPDH as described

## SUPPLEMENTARY MATERIAL FIGURES AND TABLES


