# Supplementary material for: A class of genes in the HER2 regulon that is poised for transcription in breast cancer cell lines and expressed in human breast tumors
Source: Oncotarget. 2014 Nov 4;6(2):1286–301. doi: 10.18632/oncotarget.2676 (PMC4359233; doi:10.18632/oncotarget.2676)
Supplement: Supplementary file 1 [file oncotarget-06-1286-s001.pdf]

A class of genes in the HER2 regulon that is poised for transcription in breast cancer cell lines and expressed in human breast tumors

Supplementary Material

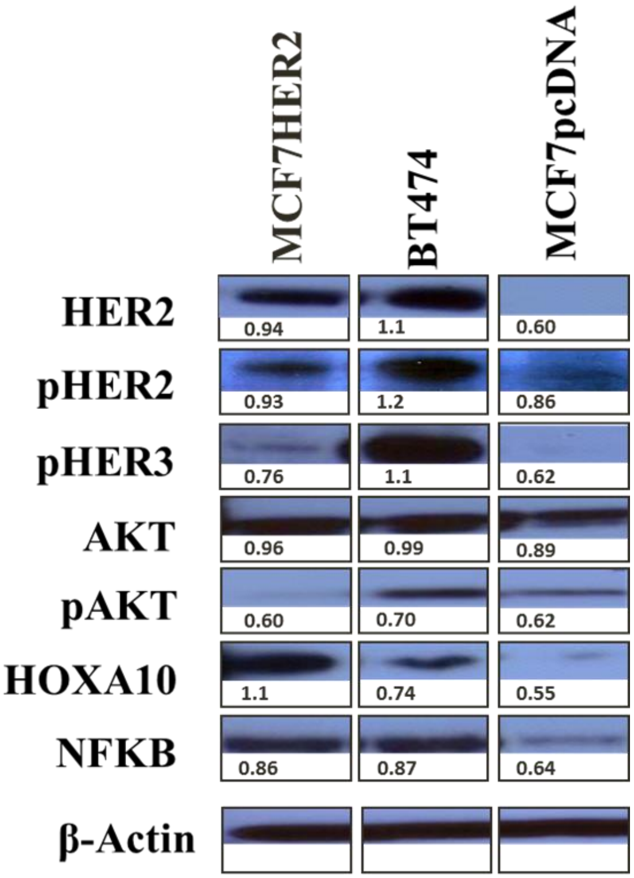

Figure S1: Western blotting to estimate protein expression. We examined MCF7 cells with and without stable expression of HER2 termed MCF7PHER2 and MCF7pcDNA cells. MCF7HER2 cells stably over express HER2 to levels comparable to BT474, a human breast cancer cell line with known high levels of amplified and expressed HER2. In contrast, immuno-reactive HER2 was undetectable in the MCF7pcDNA control cells. In order to confirm that over-expressed HER2 was functional, we examined the level of phosphorylation of HER2 and known downstream HER2-activated targets, HER3, HOXA10 and NFκB . HER2 was observed to be constitutively phosphorylated in agreement with our previously published observations [1] . In addition we observed increased phosphorylation of HER3 heterodimer partner of HER2 which was not observed in MCF7pcDNA cells as well as HoxA10 and NFκB . These results indicate that cells used here reliably exhibit expression and signal transduction features of HER2 signaling. Relative expression was determined using densitometry (Alpha Ease FC™ (Alpha Innotech Corporation) imager software). The average signal (sum of pixels/area) for the protein of interest was calculated and compared to the average signal detected for the housekeeping gene β-*actin*.

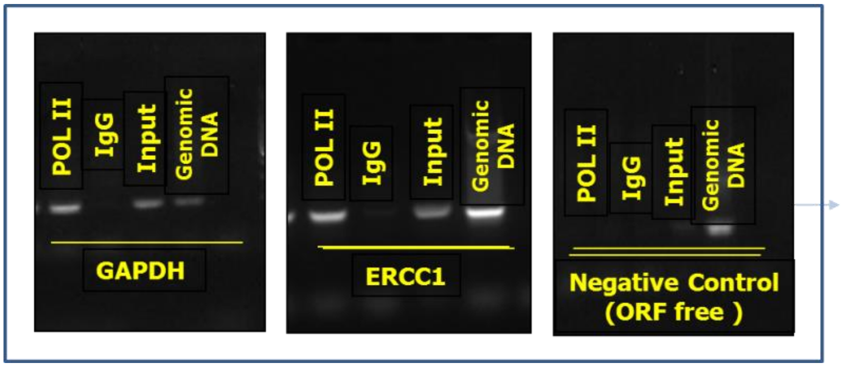

Figure S2 (A): Detecting RNA POL II enrichment at GPDH, ERCC1 and Open Reading Frame (ORF in MCF7HER2. We used 40 cycles of PCR on 10 ng of captured material to analyze the specific enrichment of ChIP-captured DNA in all examined breast cancer cell lines (MCF7HER2 is shown as an example) for the GAPDH, ERCC1 (known POL II target genes) and for an ORF-Free region as a negative control. The PCR products were resolved using 2% agarose gels.

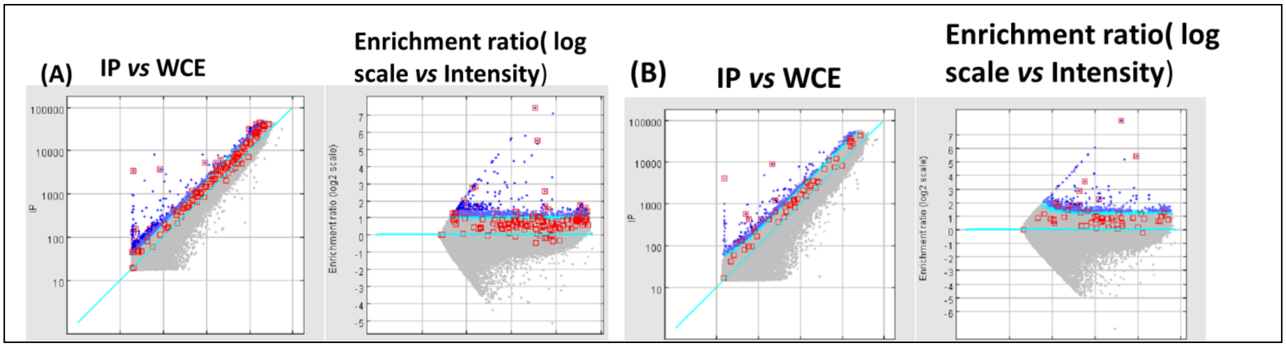

Figure S2 (B): ChIP-chip analysis of POL II in BT474, high HER2 expressing breast cancer cells. ChIP-chip analysis was performed using a human Agilent promoter arrays with 500K probe sets, **A** and **B**. Feature extraction software (Agilent) and DNA analytic software (ChIP module, Agilent) was used to determine bound regions in the dataset by using the Whitehead Per-array neighborhood model . The scatter plots are screen shots taken from QC reports from DNA Analytics v. 4.0 of Genome Work Bench from Agilent. The scatter plots of **(A)** and **(B)** show POL II enriched intensity (processed IP signal) relative to whole cell extract for all genes of Agilent promoter array. BT474 is shown as an example. The features are significantly enriched in POL II precipitated DNA are shown in red and blue where the red and blue dots represent POL II binding with $0.001 < p < 0.05$ and $p < 0.001$ respectively. Y and X axis represent the RNA POL II enriched signal intensity (IP) and whole cell extract signal intensity (WCE) respectively.

(A)

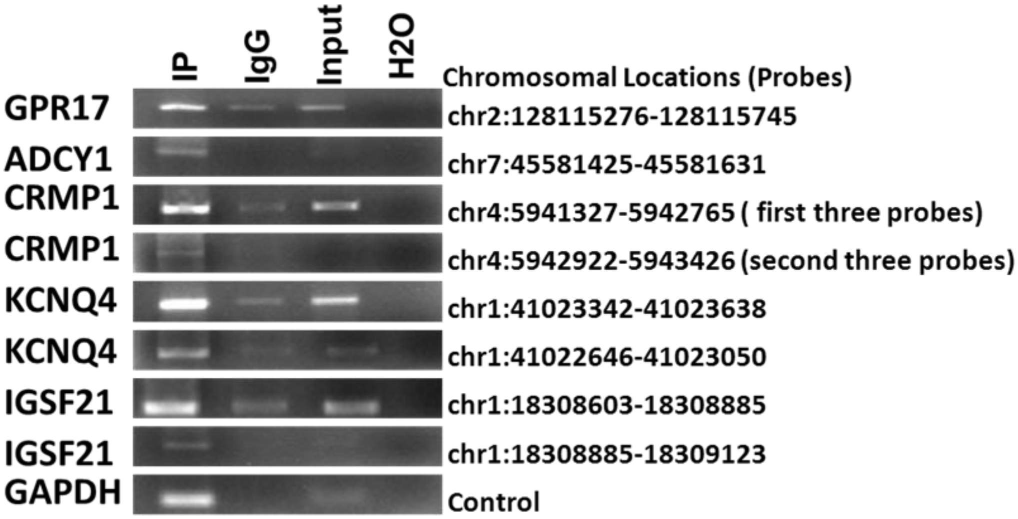

(B)

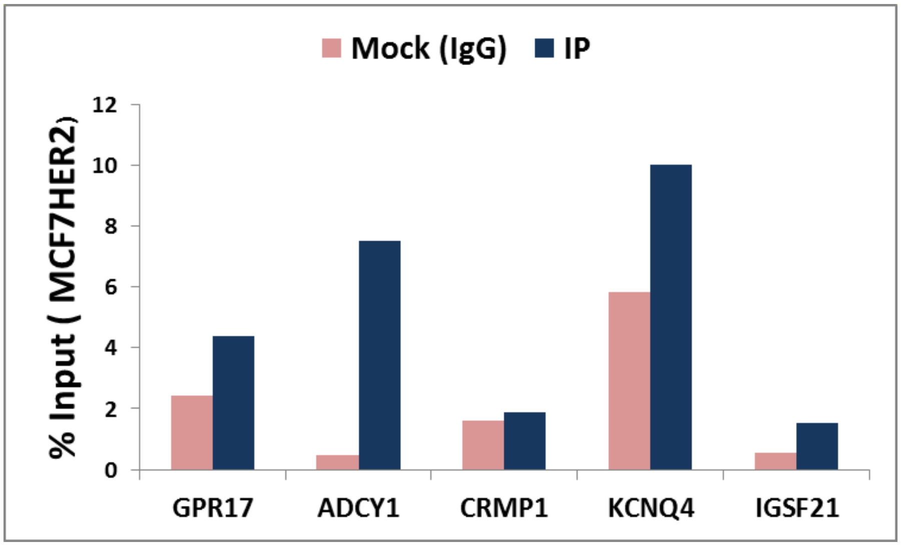

Figure S3: Validation of POL II binding in MCF7HER2 cells. POL II binding sites in the ChIP captured DNA of MCF7HER2 cells were validated by utilizing Agarose gel based ChIP-PCR (Panel A) and quantitative real time ChIP-PCR (Panel B). In panel B the direct binding of POL II with the promoters of GPR17, ACDY1, CRMP1, KCNQ4 and IGSF21 is illustrated. The percent of input represents the % of DNA that is precipitated by POL II. Non-immune antibody IgG was used as negative control.

Table S1: Intersection of POLII binding data for MCF7HER2, MDA453 and BT474

**MCF7HER2 *vs* pcDNA**	**Number of POL II bound genes (*p* < 0.05)**	**Number of POL II bound Genes (0.05 < *p* < 0.13)**
**MCF7HER2 -unique**	606	1638
**MCF7pcDNA-unique**	678	1504
**Common**	1079	11045

**BT474 *vs* pcDNA**	**Number of POL II bound genes (*p* < 0.05)**	**Number of POL II bound Genes (0.05 < *p* < 0.13)**
**BT474-unique**	5115	2801
**MCF7pcDNA-unique**	842	7064
**Common**	915	5485

**MDA453 *vs* pcDNA**	**Number of POL II bound genes (*p* < 0.05)**	**Number of POL II bound Genes (0.05 < *p* < 0.13)**
**MDA453-unique**	2149	2464
**MCF7pcDNA-unique**	821	3963
**Common**	936	8586

The total number of POL II bound genes in high HER2 expressing cells was compared to those of non-expressing HER2 cells (*e.g.,* MCF7pcDNA). The number of uniquely bound POL II genes as well as the number of common genes for each pairwise comparison is shown. Tightly bound POL II genes is indicated as $p < 0.05$, whereas $0.05 < p < 0.13$ indicates number of loosely bound POL II genes. Comparison of the distribution of binding of POL II in MCF7HER2 cells to control cells revealed a striking patterns of rearrangement associated with the expression of HER2 consisting of binding to new genes of the HER2-expressing cells and loss of binding to previously bound genes of the control cells. For example, 678 POL II-binding genes in MCF7pcDNA control cells no longer bound POL II in MCF7HER2 cells. In contrast in HER2-expressing cells exhibited 606 other genes that *gained* POL II binding indicating a very substantial shift in the localization of POL II upon the stable expression of HER2. In addition changes in the number of sites bound per gene for genes *common* to the two cell types were also identified.

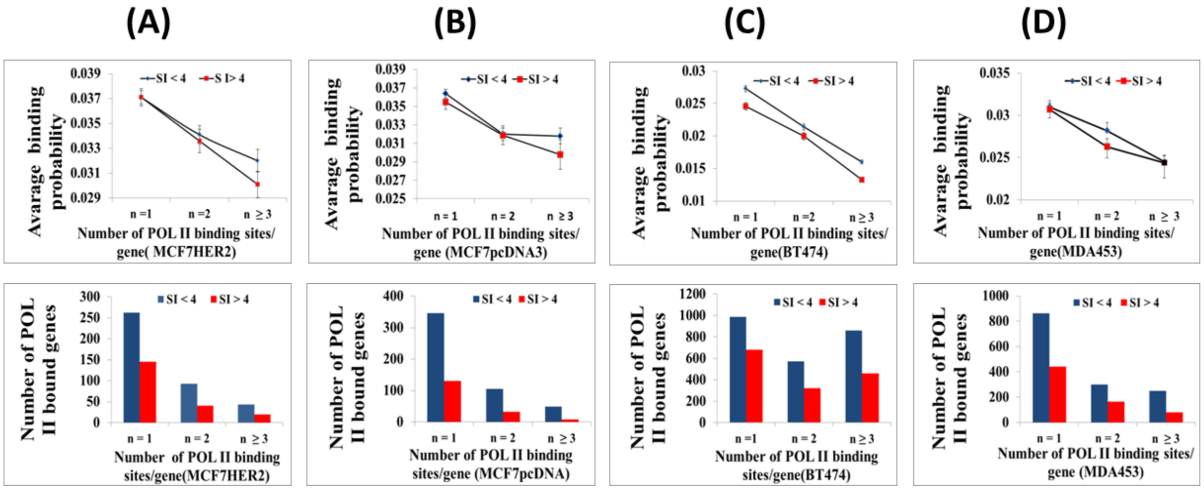

Figure S4: POL II binding probability as function of number of binding sites and position in the gene. For each cell line we calculated the stalling index (genes selected based on *p* value binding < 0.05) then ask whether the multiple POL II binding sites in the gene is associated with both "tight binding" (low *p* value) and location. The analysis is for all significantly bound genes of a cell line. **(A)** and **(B)** MCF7HER2 (606 and 678 genes gained and lost POL II binding sites (all with $p < 0.05$) upon HER2 expression, respectively. The relationship between averaged *p* value, the number of binding sites/gene and location for the unique POL II binding genes of **(C)** BT474($p < 0.05$) and **(D**) MDA453($p < 0.05$) are also summarized. Those genes that exhibit significant POL II binding in the MCF7pcDNA cells were excluded in order to eliminate overlapping gene identities. The error bars are s.e. values for the variation of *p* values. Each bar graph represents the number of genes of each group (SI < 4 and SI > 4). POL II bound genes were divided into three groups based on their number of binding sites/gene where the number is 1, 2, or ≥ 3 (x-axis). There are relatively few genes with number ≥ 3 and the probabilities for these cases were combined to form "average" probability per site as described in the Materials and Methods. The binding probabilities, *p*, decline as the binding number increases from 1 to 2 to 3 or more sites per gene indicating that the tightness of binding is correlated with increased number of sites for a given gene. The relationship between the probability of binding and the number of POL II sites/gene is independent of HER2 since a similar trend holds for the 678 significant POL II-bound genes of the MCF7 control cells with different gene identities. Genes with multiple binding sites also tend to cluster near the TSS (SI > 4) whereas genes with higher values tend to have few binding sites/gene which are found downstream (SI < 4). These results identify a class of looser bound more mobile genes located in the 3' coding region. Our results illustrate a large effect of HER2 overexpression in shifting the POL II binding site (accumulation) toward a more 3' coding region.

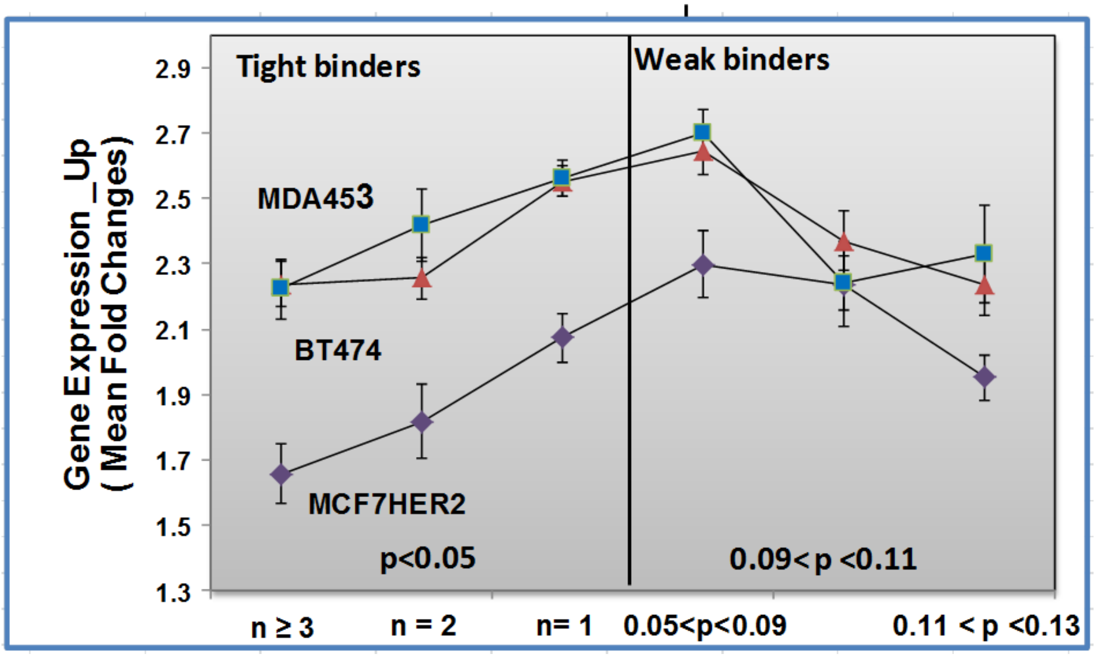

Figure S5: Expression as FC of the 3 high HER2-expressing cell lines relative to MCF7pcDNA control cells as function of POL II binding probability. Expression (up- regulated genes) persists or increases in the binding probability range 0.05<*p*<0.13 and declines at low *p*.

Table S2: 51 transcripts differentially expressed in the same direction in all high HER2 expressing cell lines *vs* non HER2 expressing cells**.**

Probe ID	Gene Name	MCF7 HER2 Log2 FC	BT474 Log2 FC	MDA453 Log2 FC	HER2+/ Primary tissue Log2 FC	*P*-Value
225185_at	**MRAS**	**-1.14**	**-0.53**	**-1.11**	**-0.51**	**9.74E-10**
209648_x_at	**SOCS5**	**-1.13**	**-1.18**	**-0.56**	**-0.46**	**5.07E-08**
203853_s_at	**GAB2**	**-0.79**	**-2.15**	**-3.09**	**-0.39**	**3.06E-06**
222557_at	**STMN3**	**-0.6**	**-0.96**	**-4.19**	**-0.22**	**0.007798**
32541_at	**PPP3CC**	**-0.36**	**-0.62**	**-3.02**	**-0.17**	**0.040751**
35666_at	**SEMA3F**	**0.95**	**0.56**	**2.07**	**0.37**	**1.12E-05**
202201_at	**BLVRB**	**0.99**	**1.04**	**1.69**	**0.33**	**9.39E-05**
200635_s_at	**PTPRF**	**0.34**	**0.9**	**0.9**	**0.25**	**0.003308**
201669_s_at	**MARCKS**	**4.3**	**4.62**	**3.19**	**0.23**	**0.005547**
218760_at	**COQ6**	**0.62**	**0.39**	**1.01**	**0.2**	**0.019316**
210240_s_at	**CDKN2D**	**1.04**	**1.42**	**2.1**	**-0.17**	**0.03928**
233054_at	**CNOT2**	**-0.78**	**-1.13**	**-1.18**	**0.32**	**0.000119**
203059_s_at	**PAPSS2**	**-1.26**	**-2.37**	**-2.23**	**0.31**	**0.000281**
219317_at	POLI	-0.58	-0.45	-0.6	0.15	0.081462
226030_at	ACADSB	-1.22	-0.97	-0.47	0.13	0.114695
236006_s_at	AKAP10	-0.4	-1.01	-0.76	0.13	0.121644
220261_s_at	ZDHHC4	-0.73	-0.74	-1	-0.12	0.153333
204183_s_at	ADRBK2	0.53	0.54	0.77	-0.12	0.155393
202304_at	FNDC3A	1.25	0.6	1.81	-0.11	0.186701
204639_at	ADA	-2.02	-1.2	-3.15	-0.11	0.20604
228674_s_at	EML4	-1.17	-1.09	-1.34	-0.07	0.393778
226914_at	ARPC5L	1.03	1.56	0.82	0.06	0.466863
238034_at	CANX	-0.45	-0.45	-0.48	-0.06	0.481918
219201_s_at	TWSG1	-1.28	-1.43	-1.81	-0.04	0.596443
200731_s_at	PTP4A1	-0.46	-0.9	-0.98	-0.03	0.722849
201209_at	HDAC1	-0.47	-1.61	-1.44	-0.03	0.746337
200820_at	PSMD8	0.64	0.55	0.7	0.02	0.777463
206744_s_at	ZMYM5	-0.63	-2.01	-1.79	-0.01	0.917904
228391_at	CYP4V2	-2.73	-1.74	-2.11	-	-
230769_at	DENND2C	-2.36	-4.38	-4.34	-	-
242138_at	DLX1	-0.98	-2.64	-1.15	-	-
207147_at	DLX2	-1.21	-1.81	-1.71	-	-
238823_at	FMNL3	-0.47	-0.66	-0.7	-	-
216442_x_at	FN1	-1.96	-5.2	-5.29	-	-
230645_at	FRMD3	-0.87	-1.9	-3.1	-	-
225571_at	LIFR	-3.59	-2.18	-4	-	-
203466_at	MPV17	-0.72	-1.96	-1.43	-	-
215228_at	NHLH2	-0.87	-1.22	-1.11	-	-
242123_at	PAQR7	-1.03	-1.62	-1.58	-	-
204944_at	PTPRG	-0.96	-2.04	-2.05	-	-
228497_at	SLC22A15	-1.8	-2.81	-4.81	-	-
200991_s_at	SNX17	-0.53	-0.77	-0.34	-	-
226186_at	TMOD2	-1.37	-1.56	-2.09	-	-
205586_x_at	VGF	-0.9	-1.06	-1.11	-	-
209989_at	ZNF268	-0.67	-0.84	-0.59	-	-
1553132_a_at	MTAC2D1	0.93	1.58	1.93	-	-
212867_at	NCOA2	0.82	1.08	1.2	-	-

(-): None Existence; genes that did not appeared among the most significant 3350 (all $p < 0.05$) genes from primary tissue datasets.

Table S3: Validations of microarray data by real time quantitative PCR: Comparison of Affymetrix data with RT-PCR

Gene Name	MCF7HER2 qPCR (log2FC)	MCF7HER2 Affymetrix (log2FC)	BT474 qPCR (log2FC)	BT474 Affymetrix (log2FC)	MDA453 qPCR log2(FC)	MDA453 Affymetrix (log2FC)
**ALDH4A1**	-1.286	-0.418	1.885	1.528	3.394	2.581
**CCNG2**	-0.312	-	4.108	2.689	2.290	1.630
**CRIP1**	-0.982	0.747	8.759	7.573	3.511	2.816
**CXXC1**	1.013	0.601	4.708	1.265	5.024	1.374
**DIRAS2**	-0.701	1.094	-2.617	-2.473	-11.452	-3.263
**DKK1**	-0.445	1.351	-2.421	-3.432	-8.057	-7.468
**DNMT3A**	0.692	1.211	1.373	0.465	1.990	0.844
**ESRRG**	-0.034	-	8.919	4.025	7.242	0.578
**GAD1**	-0.732	-1.984	-0.845	-2.143	-4.991	-2.147
**GPX3**	0.061	-2.053	-5.426	-5.863	-8.008	-5.827
**IRAK4**	-0.574	-0.837	1.435	-1.488	-0.380	-1.568
**IRX4**	-0.340	-	3.384	2.991	0.631	0.595
**KRT7**	-0.182	-0.969	9.986	4.376	8.480	0.458
**MPV17**	-0.691	-0.722	-0.556	-1.961	-0.399	-1.429
**MRAS**	-0.528	-1.138	-0.391	-0.533	-1.220	-0.623
**PCDHAC1**	-0.268	-0.529	0.714	-3.116	-1.639	-0.203
**PEG10**	3.541	-2.278	4.244	2.638	10.207	2.358
**PIP5K1B**	0.255	1.855	4.701	2.490	2.689	1.381
**PKIB**	0.130	-0.665	-0.860	-1.757	-2.370	-4.399
**PMP22**	-0.529	-0.916	-4.427	-4.583	-5.092	-5.150
**RARRES1**	-1.365	-	5.405	4.221	5.123	1.221
**RASD1**	0.683	-	-2.985	-2.732	-3.914	-2.606
**S100A14**	0.741	1.982	4.260	2.119	4.148	2.722
**SEMA3F**	-0.247	0.592	1.898	0.565	3.024	1.487
**SOCS5**	-0.647	-0.732	-0.318	-1.073	0.336	-0.517
**STMN3**	0.027	-0.596	0.532	-0.962	-3.253	-4.193
**TNC**	-1.422	-	10.319	5.923	12.731	1.422
**TRPC1**	0.220	-0.687	-3.026	-1.530	-7.271	-3.663
**VGF**	0.814	-0.897	-4.414	-1.057	-4.351	-1.113
**ZDHHC11**	-1.302	-	5.579	2.955	2.432	1.181
**ADCY1**	-0.161	0.478	-3.110	-3.838	-9.929	-5.905
**CELSR2**	-0.653	0.671	-1.940	-3.143	-3.031	-2.564
**RGS12**	-0.536	0.388	0.352	-	0.385	0.425
**LCN2**	0.510		2.901	-	-5.468	-0.414
**LAMA5**	0.093	0.599	0.097	-0.819	1.347	-
**CNOT3**	0.483		1.126	-0.530	1.356	-
**MUC1**	2.384	4.181	4.072	2.762	-0.947	-0.273
**DDX41**	-0.371	0.494	0.450	-	1.021	-4.937
**PAX2**	2.907	-	5.359	1.202	5.293	-1.194
**TCEA3**	-0.984	-1.579	0.263	-0.576	4.111	0.569
**GABRP**	8.645	-	7.502	-	0.886	-
**BTG3**	9.639	3.296	15.172	6.059	15.767	5.361
**MARCKS**	0.741	4.725	5.730	5.267	2.576	0.846

**STXBP4**	0.626	1.815	6.211	5.175	3.866	1.109
**MYRIP**	1.964	3.901	6.531	3.738	6.103	3.221
**PRSS8**	1.396	3.751	5.088	3.188	4.745	3.771
**EFHD1**	2.644	5.123	8.511	6.038	6.646	4.728
**ZNF22**	6.052	4.518	10.677	4.887	11.271	6.293
**CYBA**	10.814	5.501	11.639	3.776	16.128	5.105
**SHC1**	0.459	0.332	1.271	-	0.904	-
**SIAH1**	-0.262	-0.475	1.317	0.582	0.615	0.541

Genes were selected for validations by qPCR if they were regulated in the same direction with the *p* value < 0.05 in at least 2 of the 3 high HER2 expressing cells relative to control non expressing HER2 cells (*i.e.*, MCF7pcDNA3) shown by Affymetrix gene expression data. All Cycle threshold (Ct) values of the candidate genes were normalized to housekeeping gene, *i.e.*, GAPDH. The fold changes in gene expression between the high and low HER2 expressing cells was calculated as $2^{(-\Delta\Delta Ct)}$. Affymetrix values are indicated. Pearson Correlation Coefficients between the Affymetrix values and the qPCR values are about 0.74 – 0.89 for all three cell lines.

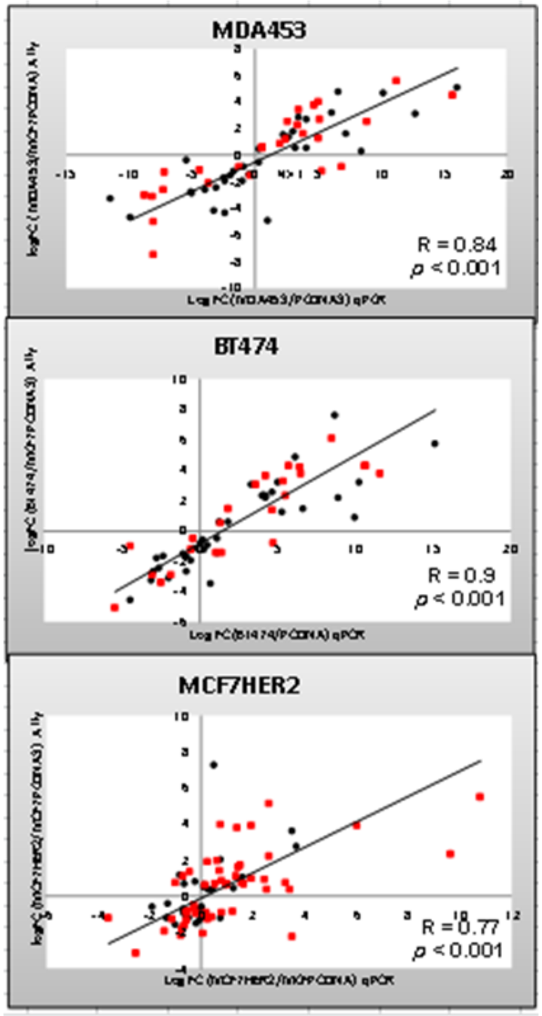

Figure S6: Comparison of Affymetrix data with RT-PCR using the same RNA preparation for the three high HER2-expressing lines. We validated the expression data for representative genes taken from the up- and down-regulated transcripts of all classes and cell lines including the genes with low POL II binding ($0.05 < p < 0.13$) shown as red and those with high binding ($p < 0.05$) shown as black by qPCR. Pearson Correlation Coefficients between the Affymetrix values and the qPCR values are about 0.74 – 0.89 for all three cell lines.

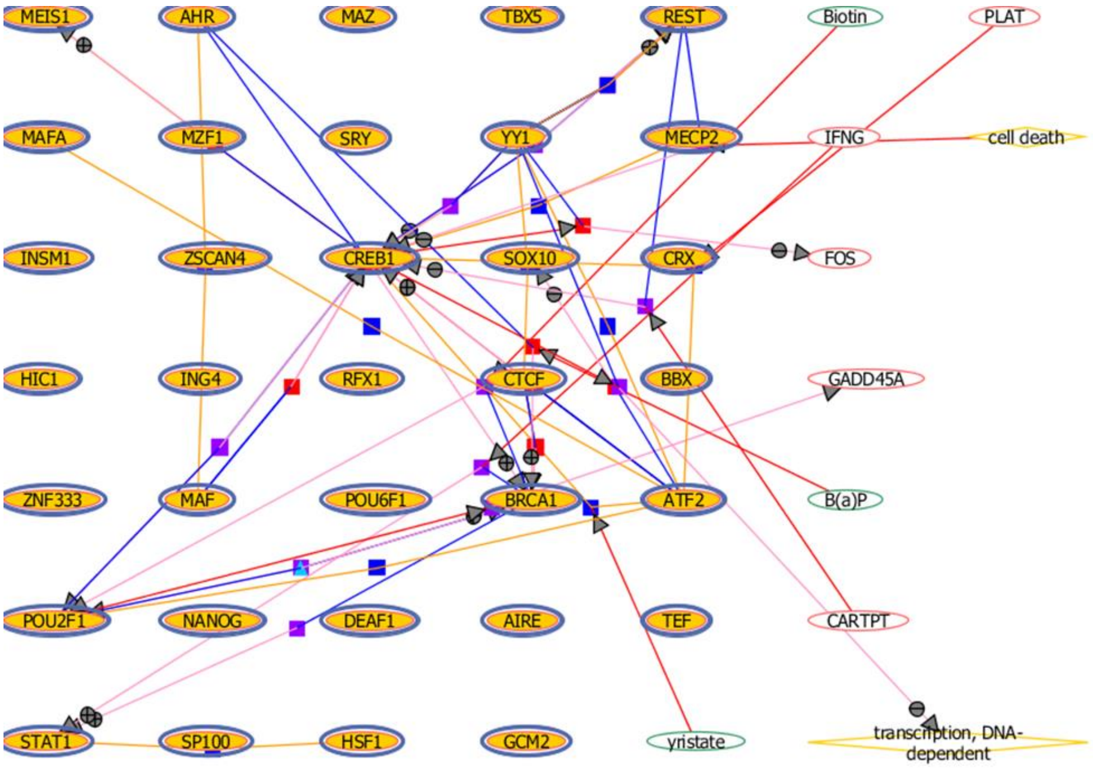

Figure S7: Network analysis (Strand-NGS pathway analysis tool, Agilent) on the sets of predicted transcription factors associated with the regulatory element sequences from MATCH [TM] analysis of 113 tissue context dependent genes. Insert image shows the direct interactions among the TFs (highlighted brown color).TFs without direct interaction are eliminated.

Figure S8: HER2 expression increases the expression levels of ALDH1A1, NANOG, SOX and OCT3/4 in cultures of “mammospheres” of MCF7HER2 cells compared to attached cultures and to MCF7pcDNA3 cells.

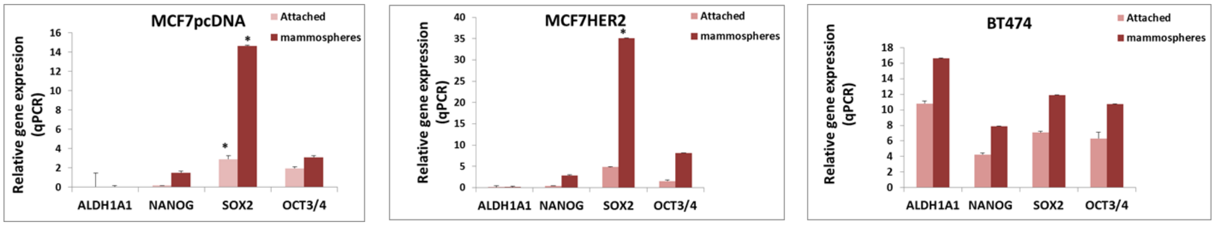

Expression levels of ALDH1A1, NANOG, SOX2 and OCT3/4 was quantified using real time RT-PCR in attached culture of MCF7pcDNA, MCF7HER2 and BT474 as well as mammospheres of the same cells. GAPDH (housekeeping gene) was used as an internal control. The relative expression was determined using $2^{\Delta\Delta Ct}$ method. The value represents the mean s.e. of the technical replicates. (*) the original expression level is 10 fold higher than shown here.

Table S4: The sequences for amplifying NANOG, ALDH1A1, OCT3/4 and SOX2 is shown.

	Forward	Reverse
**NANOG**	CCTTCTGCGTCACACCATT	AACTCTCCAACATCCTGAACC
**ALDH1A1**	GTTCTTCTGAGAGATTTTCACTGTG	TGGTGGATTCAAGATGTCTGG
**OCT3/4 (POU5F1)**	TGTGTCTATCTACTGTGTCCCA	GTTGGAGGGAAGGTGAAGTTC
**SOX2**	CTTGACCACCGAACCCAT	GTACAACTCCATGACCAGCTC

Supplementary information:

Protein Expression Analysis, Western blotting

In order to examine the HER2-dependendence of POL II chromatin binding in breast cancer cells we utilized MCF7 cells that are devoid of HER2 expression but which were engineered to constitutively express large amounts of HER2, MCF7HER2 cells, as previously described [1]. We confirmed that the cells grown for these studies retained characteristic properties such as stable expression of HER2 by western analysis (**Figure S1**)**.** We also examined two naturally occurring human breast cancer cell lines, BT474 and MDA453, which were originally derived from HER2-positive breast cancers that exhibited amplified HER2 [3, 4]. The HER2-expressing MCF7 cells termed MCF7HER2, BT474 and MDA453 induce increased expression of large amounts of HER2 and in addition increased EGFR, pHER3 and especially HER4 [5]. The three high HER2 expressing cells exhibit moderate amounts of phosphorylated HER2 and Akt whereas these forms are undetectable in control cells as previously reported [1].Thus these observations are in agreement with the known expression of functional HER2 [1, 6-9] and confirm that the cells used here are appropriate models for HER2 for the goals of this study.

Agilent Human Promoter Array and data analysis

Chip-chip with anti-POL II-precipitated DNA was used to identify the genes bound by POL II in mid log phase MCF7HER2 and the empty vector MCF7control cells. 5758 significantly ($p < 0.05$) bound probe sets corresponding to 2363 genes of the ~17,000 genes represented on the array were identified. Comparison of the distribution of binding of POL II in MCF7HER2 cells to control cells revealed a striking patterns of rearrangement associated with the expression of HER2 consisting of binding to new genes of the HER2-expressing cells and loss of binding to previously bound genes of the control cells. For example, 678 POL II-binding genes in MCF7pcDNA control cells no longer bound POL II in MCF7HER2 cells. In contrast in HER2-expressing cells exhibited 606 other genes that *gained* POL II binding indicating a very substantial shift in the localization of POL II upon the stable expression of HER2. In addition changes in the number of sites bound per gene for genes *common* to the two cell types were also identified. The number of sites/gene, n, was commonly altered both by gain and loss, Δn, of sites

upon expression of HER2. Up to 10 significantly bound sites/gene were observed. In all, 1079 of the 2363 common POL II-bound genes (33.8%) exhibit a change in number of POL II sites/gene upon expression of HER2 with 19% exhibiting a gain of sites and 14.8% exhibiting a decrease in binding number/gene. In order to examine further the generality of this effect we carried out Chip-chip studies of the two additional human breast cancer cells lines, BT474 and MDA453 cells. These cells also exhibit large numbers of significant POL II binding genes, 5115 and 2149 (**Table S1 & Figure S4**).

POL II binding and Stalling Index

The region examined by the Agilent arrays covers a zone of -5000 to +2500 bps with respect to the TSS, since low binding probability is reflection of high abundance of POL II-DNA complex formation the correlation may indicate the regulation of affinity of interaction extends over a large range. In order to define the relationship between binding affinity as indicated by *p- value of bindings* and location within a gene, the "stalling index", SI, of Young and co-workers[10] with slight modification was adopted. SI is the fraction of occupied probe sets of a gene located within $\pm$ 1kb of the TSS relative to the total number of occupied probe sites (**Table S1 and Figure S4**).

References for the Supplement

1. Mitra D, Brumlik MJ, Okamgba SU, Zhu Y, Duplessis TT, Parvani JG, Lesko SM, Brogi E, Jones FE: **An oncogenic isoform of HER2 associated with locally disseminated breast cancer and trastuzumab resistance**. *Molecular Cancer Therapeutics* 2009, **8**(8):2152-2162.
2. Mitra AB, Murty VVVS, Pratap M, Sodhani P, Chaganti RSK: **ERBB2 (HER2/neu) Oncogene Is Frequently Amplified in Squamous Cell Carcinoma of the Uterine Cervix**. *Cancer Research* 1994, **54**(3):637-639.
3. Lasfargues EY, Coutinho WG, Dion AS: **A Human Breast Tumor Cell Line Bt-474 That Supports Mouse Mammary Tumor Virus Replication**. *In Vitro (Rockville)* 1979, **15**(9):723-729.
4. Cailleau R, Cruciger QVJ, Olive M: **Long-Term Human Breast Carcinoma Cell Lines of Metastatic Origin Preliminary Characterization**. *In Vitro (Rockville)* 1978, **14**(11):911-915.
5. Vidal GA, Clark DE, Marrero L, Jones FE: **A constitutively active ERBB4/HER4 allele with enhanced transcriptional coactivation and cell-killing activities**. *Oncogene* 2007, **26**(3):462-466.
6. Kirkegaard T, Witton CJ, McGlynn LM, Tovey SM, Dunne B, Lyon A, Bartlett JMS: **AKT activation predicts outcome in breast cancer patients treated with tamoxifen**. *Journal of Pathology* 2005, **207**(2):139-146.
7. Gallagher RI, VanMeter AJ, Jones H, Wescott L, Sweetman RW, Petricoin EF, Liotta LA, Espina V, O'Shaughnessy J, Holmes FA: **Mapping tyrosine kinase signaling pathways in microdissected breast cancer before and after monotherapy or combination therapy with Lapatinib or Trastuzumab**. *Proceedings of the American Association for Cancer Research Annual Meeting* 2009, **50**:665.
8. Mackay A, Jones C, Dexter T, Silva RLA, Bulmer K, Jones A, Simpson P, Harris RA, Jat PS, Neville AM *et al*: **cDNA microarray analysis of genes associated with ERBB2 (HER2/neu) overexpression in human mammary luminal epithelial cells**. *Oncogene* 2003, **22**(17):2680-2688.
9. Jones FE, Stern DF: **Expression of dominant-negative ErbB2 in the mammary gland of transgenic mice reveals a role in lobuloalveolar development and lactation**. *Oncogene* 1999, **18**(23):3481-3490.
10. Zeitlinger J, Stark A, Kellis M, Hong J-W, Nechaev S, Adelman K, Levine M, Young RA: **RNA polymerase stalling at developmental control genes in the Drosophila melanogaster embryo**. *Nature Genetics* 2007, **39**(12):1512-1516.
